# Intraoral Scanning for Monitoring Dental Wear and Its Risk Factors: A Prospective Study

**DOI:** 10.3390/healthcare12111069

**Published:** 2024-05-24

**Authors:** Víctor Díaz-Flores García, Yolanda Freire, Susana David Fernández, Margarita Gómez Sánchez, Beatriz Tomás Murillo, Ana Suárez

**Affiliations:** 1 Department of Pre-Clinical Dentistry, School of Biomedical Sciences, Universidad Europea de Madrid, Calle Tajo s/n, Villaviciosa de Odón, 28670 Madrid, Spain; victor.diaz-flores@universidadeuropea.es (V.D.-F.G.); ana.suarez@universidadeuropea.es (A.S.); 2 Department of Clinical Dentistry, School of Biomedical Sciences, Universidad Europea de Madrid, Calle Tajo s/n, Villaviciosa de Odón, 28670 Madrid, Spain

**Keywords:** intraoral scanner, image diagnosis, tooth wear, etiological factors, abrasion, erosion

## Abstract

Dental wear arises from mechanical (attrition or abrasion) and chemical (erosion) factors. Despite its prevalence and clinical significance, accurately measuring and understanding its causes remain challenging in everyday practice. This one-year study with 39 participants involved comprehensive examinations and full-arch intraoral scans at the start and after 12 months. Volume loss exceeding 100 µ on each tooth’s surfaces (buccal, lingual/palatine and incisal/occlusal) was measured by comparing three-dimensional scans from both time points. This study also assessed factors such as abrasion and erosion through clinical exams and questionnaires. There were no significant differences in dental wear in participants with sleep bruxism. However, noticeable wear occurred in the front teeth of those with waking bruxism and joint-related symptoms. Increased wear was associated with frequent consumption of acidic drinks, regular swimming, dry mouth, nocturnal drooling and heartburn, while no significant wear was found in patients with reflux. The used methodology proved effective in accurately assessing the progression of dental wear, which is important as many patients may initially be asymptomatic. The variability observed in dental wear patterns underscores the need to develop specific software applications that allow immediate and efficient comparison of wear areas based on extensive analysis of patient databases.

## 1. Introduction

Dental wear, defined as the irreversible loss of hard dental tissue in the absence of bacterial action or dental trauma [1,2,3], has a multifactorial aetiology [4]. It can be classified according to its mechanical or chemical origin [5] and further subdivided into intrinsic and extrinsic categories [6,7].

Attrition, known as intrinsic mechanical wear, is caused by tooth-to-tooth contact during chewing or by bruxism. The latter may occur during sleep (sleep bruxism), characterised by masticatory activity alternating between rhythmic (phasic) and non-rhythmic (tonic) phases, or during wakefulness (awake bruxism), where the individual is awake and there is sustained tooth contact, which may or may not be accompanied by jaw thrusting [6,8,9]. Bruxism movements have been associated with factors such as stress, anxiety [10] or psychiatric problems [11] such as depression in several population studies, particularly awake bruxism [12,13,14]. It is therefore important for dentists to understand the potential aetiology, pathophysiology and treatment strategies of bruxism [15,16], given its association with cumulative dental tissue wear over time [17,18,19].

Abrasion, or extrinsic mechanical wear, results from the frequent interposition of external objects between the teeth [20]. These objects can be hard, such as pencils, pens, nails, sunflower seeds, toothpicks, etc. [21,22,23,24], or soft, as when biting the buccal and labial mucosa [23]. Abrasion has also been reported due to inadequate washing of vegetables containing soil [24] or due to inappropriate brushing techniques or abrasive toothpastes [20,25,26].

On the other hand, erosion or chemical wear is caused by the action of non-bacterial acidic or chelating substances on the tooth surface [27,28]. An acidic environment with a pH ≤ 5.5 at the enamel surface predisposes to erosion [24], with gastroesophageal reflux disease (GERD) being one of the major intrinsic causes [3,27,29,30,31]. Extrinsic factors include exposure to acidic substances in the diet or environment [32,33] and high consumption of carbonated drinks, acidic foods, alcohol, dressings, fruit, and other foods [28,34,35,36,37,38,39]. Less common causes of dental erosion include prolonged environmental exposure to acids such as sulphuric acid and hydrochloric acid [39] and swimming in poorly chlorinated pools with a low pH and insufficient buffering system [40]. It is also important to note that some patients may have reduced salivary flow, which reduces the buffering capacity of saliva. This may be due to Sjögren’s syndrome, radiotherapy to the head and neck or the use of certain medications, making them more susceptible to dental erosion, so this condition should be considered in the risk assessment of the patient [23].

Dental wear has a high prevalence, affecting both primary and permanent teeth [41,42,43], with over 30% of the young population experiencing tooth surface volume loss, increasing with age [4].

In the context of dental wear, which is cumulative in nature, patients may either be asymptomatic or present with a wide range of clinical manifestations. These include tooth sensitivity due to exposure of dentinal tubules [24,44], aesthetic changes, loss of the vertical occlusal dimension [2,45] and disturbances in masticatory function [45]. Therefore, early diagnosis and careful monitoring of the progression of dental wear are essential [46]. This approach allows the implementation of effective preventive strategies, thereby avoiding more complex and costly treatments [47,48]. However, the clinical detection of dental tissue volume loss is a significant challenge in daily practice due to the difficulty in detecting subtle changes [4,7]. Recently, the use of intraoral scanners (IOSs) in in vivo studies has been explored as a tool for monitoring dental wear [34,46,49,50], using three-dimensional (3D) image superimposition [50]. This method has been shown to be acceptable for the measurement of dental wear [45] and is characterised by its high specificity and sensitivity [49]. Nevertheless, studies that have thoroughly investigated the factors involved in dental wear and its quantification using IOSs are still scarce [50]. In this regard, the present study focuses on quantifying dental wear using IOSs and investigating its relationship with both intrinsic and extrinsic aetiological factors.

## 2. Materials and Methods

### 2.1. Study Design

A one-year prospective clinical trial was conducted at the Dental Clinic of the European University of Madrid. The study was approved by the Regional Ethics Committee for Clinical Research of the Community of Madrid (project identification code: Desgaste-UE, 11 March 2018) and was conducted in accordance with the World Medical Association’s Declaration of Helsinki. This study was registered in Clinical Trials under ID NCT05843513. Participants were guaranteed a full understanding of the characteristics of the study to ensure their voluntary participation. Written informed consent was obtained from participants prior to their participation. They were fully informed of the aims of the study, the procedures involved, the potential risks and benefits, and their right to withdraw at any time. They were assured that all information collected would be kept confidential and used only for research purposes. Participation was voluntary and their decision would not affect their dental care. Patients received no financial compensation for their participation.

### 2.2. Patient Selection and Inclusion/Exclusion Criteria

Strict inclusion criteria were set for the study, including (i) patients over 18 years of age; (ii) those who would remain at the university for more than 18 months; and (iii) participants willing to sign an informed consent form. Although the recruitment campaign primarily targeted the university community to facilitate follow-up, participants did not necessarily have to be students. On the other hand, exclusion criteria included (i) pregnant women; (ii) patients planning to modify their oral condition through orthodontic, surgical and/or rehabilitative treatments; (iii) individuals with alterations in enamel and/or dentin development; and (iv) those with any personal or academic relationship with the study researchers.

### 2.3. Medical History and Physical Examination

Participants underwent a comprehensive medical history and detailed intraoral and extraoral examinations. Additional data on potential aetiological factors for dental wear were collected by means of a questionnaire divided into different thematic blocks. Both the examination and the questionnaire were administered at the first visit (T0) and after 12 months (T1). The items in the questionnaire were answered dichotomously (yes/no), with affirmative responses interpreted as indicative of common habits and therefore of clinical relevance. The thematic blocks of the questionnaire covered the following areas:

1 Endogenous abrasion (attrition): The use of occlusal splints, the existence of harmful oral habits and the incidence of daytime and/or nighttime grinding were considered.
2 Exogenous abrasion: Biting on hard or soft objects.
3 Exogenous erosion: Environmental factors, such as frequent swimming. Ingestion of acidic drinks or substances, such as carbonated beverages, juices, alcohol, dressings, etc.
4 Endogenous erosion: Reflux, vomiting, heartburn, nocturnal drooling, etc.

The assessment of potential bruxism was complemented with a detailed examination of the temporomandibular joint (TMJ), palpation of the muscles commonly involved in bruxism and/or TMJ disorders and the identification of the presence or absence of wear facets.

Additionally, the State Trait Anxiety Inventory (STAI) was used to measure state and trait anxiety, with scales ranging from 0 to 60 [51,52].

### 2.4. Clinical Procedure

Initially, all participants underwent bacterial plaque removal following extraoral and intraoral evaluations. This procedure was performed using a brush attached to a micromotor (KaVo Dental GmbH™, Bieberich, Germany) and prophylactic paste (Cleanic^®^, Kavo Kerr, Brea, CA, USA). In cases where dental calculus was detected, it was removed using a universal tip No. 1 (Satelec Acteon™, Merignac, France) attached to an ultrasonic device (Satelec Acteon™, Merignac, France), thus ensuring no interference with the digitization and subsequent measurement of dental wear.

Patients were then placed in an upright position with their backs straight and their heads aligned so that the occlusal plane was parallel to the floor. An Optragate^®^ mouth opener (Ivoclar Vivadent AG, Schaan, Liechtenstein) was used to retract the mucosa and a suction device to remove saliva. A fine layer of titanium oxide powder (3M Espe, St. Paul, MN, USA) was then applied to all dental surfaces of both arches using an applicator gun with a 1 mm nozzle. This layer allowed the reflected image to be captured by a lens system and projected onto a sensor. Using an intraoral scanner (3M™ True Definition intraoral scanner, 3M ESPE, Seefeld, Germany) set to active wavefront sampling mode, the buccal, lingual or palatal, and incisal or occlusal surfaces of both arches were digitized by scanning in quadrants. This initial scan, referred to as T0, was repeated after 12 months (T1).

### 2.5. Image Processing

The surfaces digitised at T0 and T1 were saved in STL format and processed using Geomagic™ Control X software (3Dsystems, Darmstadt, Germany). This processing included the use of a colorimetric scale to facilitate the visualisation of dental wear, allowing the detection of discrepancies ranging from 20 microns (µ) to 5 millimetres (mm).

**Figure 1:**
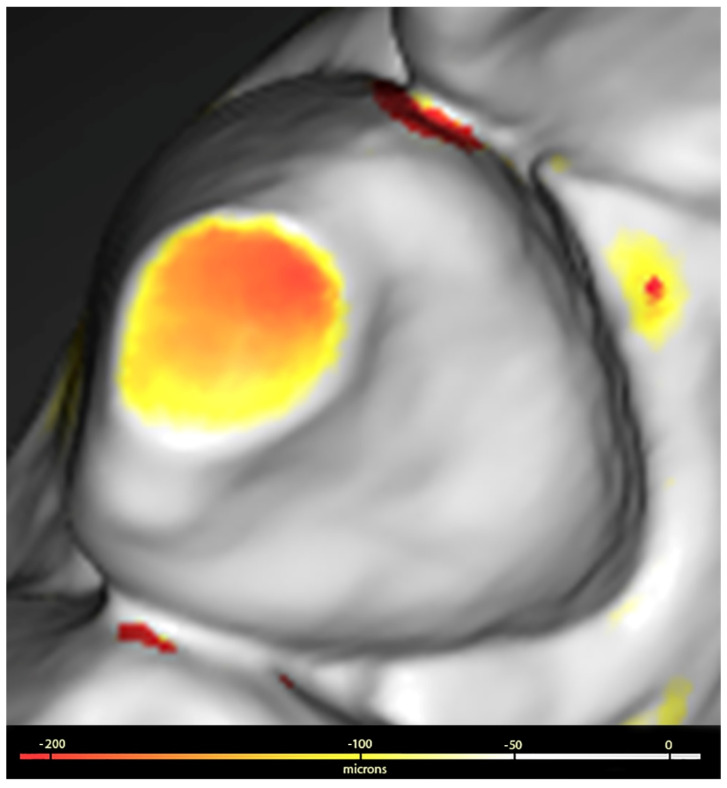
Detail of the colour signal and its correspondence in microns.

The procedure used to assess wear was as follows: the images corresponding to T0 and T1 were superimposed and aligned with Geomagic, which generated a 3D colour map that showed and quantified both the location and intensity of the volume differences on the surfaces of both sets. Volume losses (negative value) greater than 100 µ were identified on the three tooth surfaces (buccal, lingual or palatal, and incisal or occlusal). The values obtained were summed for each dental unit and for groups of teeth classified as anterosuperior (from upper canine to canine), posterosuperior (upper premolars and molars), anteroinferior (from lower canine to canine) and posteroinferior (lower premolars and molars), as shown in Figure 1.

### 2.6. Statistical Analysis

Descriptive analysis included both clinical and sociodemographic variables. For qualitative variables, absolute (n) and relative (%) frequencies were used, while for quantitative variables, mean ± standard deviation (SD) or median and interquartile range (IQR) were used, depending on the normality of their distribution.

In the inferential analysis, which aimed to study dental wear between T0 and T1, the Student’s t test or the Mann–Whitney U test were used for continuous variables, depending on their parametric nature. For qualitative variables, the Chi-square test or Fisher’s exact test was used.

All questionnaire answers and image processing data were compiled in an Excel spreadsheet (Microsoft, Albuquerque, NM, USA) and analysed using SPSS v.21.29 (IBM, SPSS Statistics, Version 21.29.0, Armonk, NY, USA: IBM Corp). Differences were considered statistically significant at *p* < 0.05.

## 3. Results

### 3.1. General Characteristics

Of the 65 participants who met the pre-defined criteria for the study, 39 agreed to participate in the study. Of these, 74.36% of the participants included in the study were male and 25.64% were female, with ages ranging from 20 to 34 years.

### 3.2. Dental Wear

The results are presented both individually for each tooth (considering its three surfaces and the total sum) and by sector (anterior and posterior). In particular, more pronounced wear was observed in the maxilla than in the mandible, as shown in Table 1 and Table 2.

### 3.3. Etiological Factors

1 Endogenous abrasion.

Table 3 shows the distribution of participants according to their self-reported symptoms related to endogenous abrasion.

The analysis revealed that patients with wear facets, nocturnal teeth grinding, clicking in the temporomandibular joint or pain on palpation of the masseter or external pterygoid muscles did not have significant levels of dental wear. Table 4 shows only the results that were statistically significant when comparing teeth with symptoms, specifying the specific surfaces.

The study also found no significant correlation between dental wear and State Trait Anxiety Inventory (STAI) scores for state and trait anxiety.

2 Possible etiological factors causing exogenous abrasion.

In relation to potential aetiological factors for exogenous abrasion, 51.28% of the participants reported engaging in parafunctional habits not related to bruxism. Of these, 90% reported biting hard objects, while 10% reported biting soft objects, as detailed in Table 5. In Table 5, only the results that were statistically significant when comparing the teeth of all patients who bit hard or soft objects are detailed, specifying the specific surfaces.

3 Potential aetiological factors for exogenous erosion.

Table 6 shows the distribution of participants who reported or denied engaging in activities that could contribute to dental wear due to exogenous erosion.

The correlation of these aetiological factors with dental wear, assessed using the Mann–Whitney U test, showed statistically significant results. In Table 7, only the results that were statistically significant when comparing the teeth of all patients with aetiological factors are detailed, specifying the specific surfaces.

4 Possible etiological factors causing endogenous erosion.

Table 8 shows the distribution of participants based on their self-reported symptoms associated with dental wear due to endogenous erosion.

In the analysis of aetiological factors, there was a notable association between nocturnal drooling and wear on the lower incisors, and between dry mouth and wear on the left lower canine. There was also a significant association between a burning sensation and dental wear on the lingual surfaces, as shown in Table 9, which details only the results that were statistically significant when comparing the teeth of all patients with aetiological factors, specifying the specific surfaces.

## 4. Discussion

The detection and monitoring of dental wear remains a challenge in clinical practice. A major obstacle in comparing studies on the relationship between potential aetiological factors and dental wear is the diversity of methodologies used, which vary between study models, intraoral photographs or a combination of both [53,54,55]. Although the Smith and Knight index [56] is widely recognised as the standard method for measuring wear, it has been criticised by some experts [57] for its limitations in differentiating wear by dentin exposure. In addition, the division of dentin exposure into thirds can lead to large variations in the quantification of wear severity, compromising the sensitivity of the results in favour of the reproducibility of the index [57]. These techniques, which often lack sensitivity, can be subjective [53]. In search of an effective solution to these challenges, recent research has validated the use of IOSs as reliable tools for monitoring and quantifying dental wear in vivo [2,34,49,50]. In this context, IOSs are emerging as tools that facilitate the implementation of an affordable and accurate wear quantification method [46]. In the present study, the use of the True Definition scanner for wear analysis was chosen due to its reliability and efficacy [58], a choice that is consistent with a systematic review published in 2023 showing that the most commonly used scanners for assessing dental wear are the True Definition, followed by the TRIOS 3, Cerec Omnicam and Planscan [59]. Furthermore, given the lack of in vivo studies [2,5,59] using this technology to quantify dental wear and its relationship to potential causes, the present study was developed.

Several methods can be used to effectively manage dental wear, including the use of splints to protect the teeth and reduce additional wear [60], medical treatments such as Botox to relax the jaw muscles and reduce the incidence of nocturnal grinding [61] and restorative materials to repair and protect teeth that are already affected and provide durable and aesthetic solutions [62].

In the assessment of dental wear attributed to endogenous abrasion, no significant association was found between nocturnal bruxism and the presence of dental wear, consistent with the findings of Bartolucci et al. [63]. However, a predominant effect on the anterior teeth was observed, particularly in participants who reported grinding during the day and in those with joint symptoms such as TMJ pain and crepitus or on palpation of the temporal or internal pterygoid muscles. Of note in this study, the presence of daytime or nighttime bruxism was subjectively determined by self-report, which requires cautious interpretation of signs of wear due to the multifactorial complexity of dental wear and its indirect implication in active bruxism [6,63]. Although bruxism has been associated in the literature with psychological problems such as anxiety and aggression [64,65,66], as well as stress at certain life stages [67,68], the relationship between sleep bruxism and general anxiety in adults remains controversial [69]. Similar to other studies, the identification of psychological factors and emotional stress was based on the STAI questionnaire, as it has reliable metric properties and is sensitive to environmental stressors [52]. However, it should be noted that it must be interpreted with caution without confirmation by sleep laboratory studies for the diagnosis of sleep bruxism [70].

The results did not show a significant relationship between dental wear and STAI scores, and there are no similar studies in the literature for comparison. Nevertheless, studies such as those by Al-Hiyasat et al. [11] and Winocur et al. [71] have found an association between dental wear and various psychiatric disorders; however, these differences should be interpreted with caution due to the nature of the group studied, which was diagnosed at the hospital level, and the methods used to measure wear.

With regard to exogenous abrasion, this type of dental wear, traditionally associated with toothbrushing, appears to be influenced more by the force applied during brushing than by the abrasive components in toothpaste [72]. Due to the complexity of measuring the brushing force applied by patients, studies in this area have mainly been carried out in vitro. In this context, our study evaluated parafunctional habits not associated with bruxism, such as biting hard and soft objects. Significant wear was observed in the anterior teeth, particularly on the entire surface, incisal edges and lingual surface. In addition, the study by Rusu Olaru et al. [22] reported cases where wear was limited to the incisal edges of anterior teeth, associated with habits such as nails or sunflower seeds. On the other hand, Nilner [73] did not observe wear in patients who bit their lips or cheeks, but did in those who bit their nails. Although it was noted that this wear occurred in the anterior teeth, no precise quantification was made.

Regarding dietary erosion, a two-year study by Schlenz et al. [50] found no correlation between tooth tissue loss and exposure to acidic substances. However, our study found more wear on certain surfaces in individuals who regularly consumed juices, energy drinks and dressings. Previous research using the Basic Erosive Wear Examination (BEWE) index to assess wear has also found an association between dental wear and the consumption of acidic foods, dairy products, fruit juices and alcohol [55,74]. Therefore, long-term studies are needed to assess the potential relationship between diet and dental wear using IOSs. Discrepancies between studies may be due to differences in dietary patterns between countries [55], as well as the methodologies used. Regarding environmental factors, our study observed increased wear on some buccal surfaces in participants who frequently swam in swimming pools, a finding consistent with previous studies [39,75] that used traditional methods to assess dental wear. Furthermore, as in our study, the most affected areas were the buccal surfaces of the incisors and canines, areas that have the most contact with pool water [39].

In this study, when investigating etiological factors potentially related to endogenous erosion, more pronounced dental wear was observed in participants who reported symptoms such as dry mouth, nocturnal drooling and heartburn. In contrast, no significant wear was found in those reporting reflux symptoms. Previous studies, such as those by Wetselaar et al. [6], have emphasised the multifactorial nature of dental wear, suggesting a possible coexistence of dry mouth and nocturnal bruxism. However, our analysis did not find a significant association between these factors. In a 2022 meta-analysis, Yanushevich et al. [30] associated dental erosion with GERD, a common gastrointestinal disorder. However, in our study, participants only reported reflux without a confirmed diagnosis of GERD, suggesting that dental wear associated with occasional reflux episodes may be lesser than that in patients with diagnosed GERD.

Regarding the methodology for analysing dental wear, some studies [76] have suggested focusing on “index teeth” to simplify the process and reduce costs. However, in our research we observed considerable variability in the wear results, possibly due to measuring each surface of each tooth and different groups of teeth. Rather than opting for simplification, it would be advisable to develop software applications that facilitate the comparison of intraoral scans and highlight surfaces with greater wear progression, using repositories of multiple scans from each patient.

The limitations of this study include the small sample size, which may limit the generalisation of the results to a larger population. In addition, the type of overbite of the patients was not taken into account, which could explain the higher wear in the anterior region in cases of deep bite and lower wear in cases of open bite. In addition, the combined interactions between factors were not considered, which may affect the full understanding of the causes of dental wear in the population studied.

Strengths of this study include the extensive use of IOSs to accurately monitor the progression of dental wear. This methodology allows for detailed and reliable monitoring, improving the ability to detect subtle changes in dental wear, which is often a challenge in daily clinical practice. In addition, this study addresses both intrinsic and extrinsic factors of dental wear, providing a comprehensive insight into the causes and progression of wear.

## 5. Conclusions

The used methodology proved effective in accurately assessing the progression of dental wear, which is important as many patients may initially be asymptomatic.

The present study also confirmed that dental wear is a multifactorial problem, influenced by both endogenous and exogenous factors. This understanding allows dental professionals to implement more specific preventive measures tailored to the individual needs of each patient, potentially improving clinical outcomes.

In addition, the findings suggest the need for improved diagnostic and monitoring tools, such as the development of advanced software capable of handling large repositories of scans and providing more accurate comparisons. This type of innovation could lead to better identification and management of risk factors associated with dental wear, allowing earlier and less invasive interventions.

## Figures and Tables

**Table 1:** Quantification of the average wear of maxillary teeth.

Upper Arch	Surface	Mean (µ)	SD
Upper right central incisor (1.1)	B	38.46	83.08
	P	33.33	65.23
	I	124.36	115.21
	Total	196.15	160.75
Upper right lateral incisor (1.2)	B	48.72	94.23
	P	66.67	84.55
	I	89.74	103.35
	Total	205.13	188.41
Upper right canine (1.3)	B	61.54	94.22
	P	69.23	102.35
	I	106.41	100.77
	Total	237.18	197.26
Upper left central incisor (2.1)	B	35.90	76.04
	P	47.44	69.73
	I	114.10	94.55
	Total	197.44	149.10
Upper left lateral incisor (2.2)	B	19.23	53.33
	P	52.56	75.17
	I	102.56	104.47
	Total	174.36	135.66
Upper left canine (2.3)	B	41.03	66.76
	P	43.59	77.95
	I	65.38	84.41
	Total	150.00	129.27
Upper Anterior Teeth (1.1, 1.2, 1.3, 2.1, 2.2, 2.3)		1160.26	535.97
1st upper right premolar (1.4)	B	51.28	104.81
	P	11.54	35.28
	O	217.95	134.01
	Total	280.77	179.04
2nd upper right premolar (1.5)	B	35.90	73.40
	P	21.79	71.45
	O	242.31	141.67
	Total	300.00	185.67
1st upper right molar (1.6)	B	83.33	115.47
	P	82.05	101.62
	O	246.15	118.87
	Total	411.54	202.76
1st upper left premolar (2.4)	B	56.41	74.50
	P	15.38	53.99
	O	211.54	149.32
	Total	283.33	157.42
2nd upper left premolar (2.5)	B	41.03	95.88
	P	14.10	49.93
	O	246.15	148.85
	Total	301.28	186.55
1st upper left molar (2.6)	B	67.95	108.51
	P	60.26	99.46
	O	276.92	109.33
	Total	405.13	208.00
Upper Posterior Teeth (1.4, 1.5, 1.6, 2.4, 2.5, 2.6)		1982.05	730.40

B: buccal; L: lingual; P: palatal; I: incisal; O: oclusal; Total: sum of P/L, B and I/O; SD: Standard Deviation.

**Table 2:** Quantification of the average wear of mandibular teeth.

Lower Arch	Surface	Mean (µ)	SD
Lower left central incisor (3.1)	B	35.90	68.78
	L	43.59	77.95
	I	78.21	83.35
	Total	157.69	139.80
Lower left lateral incisor (3.2)	B	20.51	50.94
	L	16.67	46.36
	I	70.51	73.20
	Total	107.69	84.71
Lower left canine (3.3)	B	35.90	73.40
	L	17.95	47.97
	I	67.95	77.36
	Total	121.79	121.83
Lower right central incisor (4.1)	B	30.77	64.50
	L	30.77	70.35
	I	79.49	92.99
	Total	141.03	154.69
Lower right lateral incisor (4.2)	B	42.31	89.25
	L	37.18	70.45
	I	73.08	64.73
	Total	152.56	146.43
Lower right canine (4.3)	B	48.72	99.00
	L	43.59	73.61
	I	70.51	86.39
	Total	162.82	178.71
Lower Anterior Teeth (3.1, 3.2, 3.3, 4.1, 4.2, 4.3)		843.59	490.73
1st lower left premolar (3.4)	B	37.18	74.09
	L	7.69	35.43
	O	126.92	133.70
	Total	171.79	148.14
2nd lower left premolar (3.5)	B	16.67	44.92
	L	24.36	67.74
	O	175.64	129.20
	Total	216.67	165.57
1st lower left molar (3.6)	B	47.44	80.25
	L	52.56	81.88
	O	244.87	97.19
	Total	344.87	144.09
1st lower right premolar (4.4)	B	30.77	81.61
	L	35.90	86.56
	O	165.38	102.05
	Total	232.05	183.71
2nd lower right premolar (4.5)	B	14.10	49.93
	L	34.62	86.72
	O	173.08	133.70
	Total	221.79	170.45
1st lower right molar (4.6)	B	43.59	83.65
	L	55.13	99.20
	O	256.41	135.33
	Total	355.13	188.41
Lower Posterior Teeth (3.4, 3.5, 3.6, 4.4, 4.5, 4.6)		1542.31	658.50

B: buccal; L: lingual; I: incisal; O: oclusal; Total: sum of P/L, B and I/O; SD: Standard Deviation.

**Table 3:** Presence or absence of bruxism-related symptoms.

Symptom	Yes		No	
	N	%	N	%
Teeth grinding while awake	16	41.03	23	58.97
Teeth grinding during sleep	20	51.28	19	48.72
Wear facets	31	86.11	5	13.89
Pain on palpation of the masseter muscles	7	17.95	32	82.05
Pain on palpation of the temporalis muscles	3	7.69	36	92.31
Pain on palpation of the external pterygoid muscles	8	20.51	31	79.49
Pain on palpation of the internal pterygoid muscles	11	28.21	28	71.79
Pain on palpation of the TMJ	2	5.13	37	94.87
Clicking in TMJ	14	35.9	24	64.10
Crepitus in TMJ	8	20.51	31	79.49

**Table 4:** Quantification of mean tooth wear versus bruxism-related symptoms.

			Yes		No		
Symptom	Tooth	Location	Mean Wear (µ)	SD	Mean Wear (µ)	SD	*p*-Value
Teeth grinding while awake	2.1	I	156.3	98.1	84.8	81.8	0.022 *
	4.2	I	100	48.3	54.3	68.9	0.022 *
Pain on palpation of the temporalis muscles	2.1	I	233.3	57.7	104.2	90.5	0.026 *
	1.1	B	166.7	152.8	27.8	68.1	0.019 *
	Total surfaces of 11		500	173.2	170.8	133.3	0.013 *
Pain on palpation of the internal pterygoid muscles	1.1	P	63.6	77.8	21.4	56.8	0.047 *
	4.1	I	131.8	114.6	58.9	75.8	0.044 *
Pain on palpation of the TMJ	1.1	P	150.0	70.7	27.0	59.6	0.008 *
	3.1	B	175.0	35.4	28.4	61.8	0.005 *
Crepitus in TMJ	4.1	I	162.5	130.2	58.1	68.4	0.024 *

B: buccal; L: lingual; P: palatal; I: incisal; Total: sum of P/L, B and I/O. Yes: patients who reported symptoms; No: patients who did not report symptoms. SD: standard deviation. * *p*-value < 0.05- statistically significant.

**Table 5:** Quantification of mean tooth wear vs. parafunctional habits not related to bruxism.

			Yes		No		
Etiological Factor	Tooth	Location	Mean Wear (µ)	SD	Mean Wear (µ)	SD	*p*-Value
BH	2.3	I	105.6	98.4	28.9	50.9	0.033 *
BS			50	70.7			
BH	Total surfaces of 2.3		205.6	125.9	97.4	118.4	0.031 *
BS			150	70.7			
BH	Total surfaces of 1.3		322.2	203.8	181.6	161.8	0.017 *
BS			0.0	0.0			
BH	3.1	L	50	78.6	18.4	44.8	0.012 *
BS			225	105.1			
BH	3.2	L	5.6	23.6	13.2	40.3	<0.001 *
BS			150	70.7			
BH	4.2	L	33.3	64.2	28.9	69.4	0.042 *
BS			150	70.7			
BH	4.5	B	19.4	57.2	0.0	0.0	0.030 *
BS			100	141.4			
BH	4.6	B	63.9	104.0	10.5	31.5	0.007 *
BS			175	35.4			

BH: biting hard objects; BS: biting soft objects; B: buccal; L: lingual; I: incisal; Total: sum of P/L, B and I/O. Yes: patients who reported experiencing the aetiological factors; No: patients who did not report experiencing the aetiological factors. SD: standard deviation, * *p*-value < 0.05- statistically significant.

**Table 6:** Presence or absence of etiological factors that may cause exogenous erosion.

Etiological Factor	Yes		No	
	N	%	N	%
Frequent swimming	8	20.51	31	79.49
Consumption of carbonated beverages	20	51.28	19	48.72
Consumption of energy drinks	10	25.64	29	74.36
Consumption of juice	26	66.67	13	33.33
Consumption of Alcohol	24	61.54	15	38.46
Consumption of fruit	36	92.31	3	7.69
Consumption of dressing	27	69.23	12	30.77

**Table 7:** Quantification of mean tooth wear versus etiological factors related to exogenous erosion.

			Yes		No		
Etiological Factor	Tooth	Location	Mean Wear (µ)	SD	Mean Wear (µ)	SD	*p*-Value
Frequent swimming	2.3	B	87.5	79.1	29	58.8	0.024 *
	1.1	B	93.8	82.1	24.2	78.4	0.003 *
	3.3	B	112.5	95.4	16.1	52.3	0.001 *
Consumption of energy drinks	3.2	L	50	70.7	5.2	27.9	0.004 *
	4.2	L	75	82.5	24	62.1	0.034 *
Consumption of juice	2.3	I	86.5	87.8	23.1	59.9	0.018 *
		Total surfaces of 2.3	190.4	126.5	69.2	94.7	0.004 *
	3.2	I	88.5	75.2	34.6	55.5	0.031 *
	3.4	B	55.8	85.2	0	0.0	0.011 *
		Total surfaces of 3.4	201.9	135.3	111.5	159.6	0.040 *
Consumption of dressing	1.6	B	109.3	124.8	24	62.2	0.041 *

B: buccal; L: lingual; P: palatal; I: incisal; Total: sum of P/L, B and I/O. Yes: patients who reported experiencing the aetiological factors; No: patients who did not report experiencing the aetiological factors. SD: standard deviation, * *p*-value < 0.05- statistically significant.

**Table 8:** Presence or absence of factors that may cause endogenous erosion.

Etiological Factor	Yes		No	
	N	%	N	%
Night drooling	9	23.08	30	76.92
Reflux	8	20.51	31	79.49
Dry mouth	4	89.74	35	10.26
Vomiting	0	0	39	100
Heartburn	9	23.08	30	76.92

**Table 9:** Quantification of mean tooth wear versus habits associated with exogenous erosion.

			Yes		No		
Etiological Factor	Tooth	Location	Mean Wear (µ)	SD	Mean Wear (µ)	SD	*p*-Value
Night drooling	3.1	Total surfaces of 3.1	250	156.1	130	124.3	0.031 *
	4.2	I	116.7	61.2	60	60.7	0.026 *
		Total surfaces of 4.2	272.2	195.4	116.7	108.5	0.016 *
Dry mouth	3.3	I	125	28.9	61.4	78.7	0.044 *
Heartburn	3.3	L	44.4	68.2	10	38.1	0.044 *

L: lingual; I: incisal; Total: sum of P/L, B and I/O. Yes: patients who reported experiencing the aetiological factors; No: patients who did not report experiencing the aetiological factors. SD: standard deviation. * *p*-value < 0.05- statistically significant.

## Data Availability

The data obtained in this study are available upon reasonable request.
